# ECMO Support and Operator Safety in the Context of COVID-19 Outbreak: A Regional Center Experience

**DOI:** 10.3390/membranes11050334

**Published:** 2021-04-30

**Authors:** Giorgia Montrucchio, Gabriele Sales, Rosario Urbino, Umberto Simonetti, Chiara Bonetto, Erik Cura Stura, Erika Simonato, Giovanni Fuoco, Vito Fanelli, Luca Brazzi

**Affiliations:** 1 Department of Surgical Sciences, University of Turin, 10126 Turin, Italy; gfuoco@cittadellasalute.to.it (G.F.); vito.fanelli@unito.it (V.F.); luca.brazzi@unito.it (L.B.); 2 Department of Anaesthesia, Critical Care and Emergency-Città della Salute e della Scienza Hospital—University of Turin, 10126 Turin, Italy; gsales@cittadellasalute.to.it (G.S.); rurbino@cittadellasalute.to.it (R.U.); usimonetti@cittadellasalute.to.it (U.S.); cbonetto2@cittadellasalute.to.it (C.B.); 3 Cardiothoracic Surgery Department, Division of Cardiac Surgery, Città della Salute e della Scienza, University of Turin, 10126 Turin, Italy; ecurastura@cittadellasalute.to.it (E.C.S.); esimonato@cittadellasalute.to.it (E.S.)

**Keywords:** COVID-19, severe acute respiratory syndrome coronavirus 2, pandemic, extracorporeal membrane oxygenation, personal protective equipment

## Abstract

Since the beginning of the COVID-19 emergency, the referral Intensive Care Unit for the Extracorporeal Membrane Oxygenation (ECMO) support of Piedmont Region (Italy), in cooperation with infectious disease specialists, perfusionists and cardiac surgeons, developed a protocol to guarantee operator safety during invasive procedures, among which the ECMO positioning or inter-hospital transport. The use of powered air-purifying respirators, filtering facepiece particles (FFP) 2–3 masks, protective suits, disposable sterile surgical gowns, and two pairs of sterile gloves as a part of a protocol seemed effective and feasible for trained healthcare workers and allow all the complex activities connected with the positioning of the ECMO support to be completed effectively. The simulation training on donning and doffing procedures and the presence of a dedicated team member to verify the compliance with the safety procedure effectively reassured operators and likely reduced the risk of self-contamination. From 1 March to 31 December 2020, we used the procedure in 35 severe acute respiratory distress syndrome (ARDS) patients and one acute respiratory failure caused by neoplastic total tracheal obstruction, all positive to COVID-19, to be connected to veno-venous ECMO in peripheral hospitals and centralized for ECMO management. This preliminary experience seems to confirm that the use of ECMO during COVID-19 outbreaks is feasible and the risks associated with its positioning and management are sustainable for the health-care workers and safe for patients.

## 1. Introduction

On 20 February 2020, the first COVID-19 patient was admitted to an intensive care unit (ICU) in Italy.

Pending a definitive confirmation about the effectiveness of ECMO in the context of COVID-19 infection [1,2,3,4], possible concern emerged about the possibility that ECMO positioning could be risky for healthcare workers if performed on COVID-19 patients.

Being the ICU located at ‘Città della Salute e della Scienza’ hospital in Turin (Italy) in charge of the management of severe respiratory failure in the Piedmont Region (Italy), an extraordinary consensus process was activated to revise local protocols and protective measures routinely applied by the ECMO team.

Here, we discuss the choices that have been made, the reasons underlying the adopted solutions and the experience obtained with their application.

## 2. The COVID-19 Context

At the beginning of the COVID-19 outbreak, both international and national organizations supported the plan to hospitalize suspected or confirmed severe acute respiratory syndrome coronavirus 2 (SARS-CoV-2) critical patients in contact and airborne isolation whenever possible [5,6,7].

Alternatively, patients should have been admitted to single rooms with sufficient space for operators donning and doffing [8].

These strategies, used in many facilities during the SARS pandemic, could not be applied in the context of the SARS-CoV-2 pandemic. In fact, due to the increasing number of patients, entire ICUs have to be dedicated to COVID-19 patients and others have been temporarily created and relocated outside the standard contexts.

## 3. The Protocol Preparation Phase

During previous infectious emergencies, exposed health workers developed the disease (0.6–92% and 1–10% in Ebola and Marburg viruses, respectively) mainly because of the inadequacy of personal protective equipment (PPE) and/or exposure to patients with unrecognized disease [9]. In the absence of clear evidence about the most adequate ways to protect healthcare workers during invasive procedures in patients suffering from COVID-19, a group of experts in the field of infectious emergencies was asked to review the existing literature on health protection [10].

In parallel, a multidisciplinary group involving cardiac surgeons, infectious disease specialists, intensivists, perfusionists and critical area nurses designed a training plan to allow a team of 4 experts to reach all the personnel of the hospital department possibly involved in ECMO management.

The training course developed at the end of this preparatory work involved the integration of traditional training supports (local guidelines and flow charts) with the use of simulated scenarios and practical exercises. All the personnel involved in ECMO management were subjected to a rigorous program, lasting 5 days.

## 4. Personal Protective Equipment (PPE): Existing Evidence

According to the World Health Organization (WHO) [6], European Centre for Disease Prevention and Control (ECDC) [7] and Extracorporeal Lung Support Organization (ELSO) [5], PPE to be worn in case of contact with patients with confirmed or suspected SARS-CoV-2 infection should include hair cover, fluid-resistant gown, longer sleeved gloves secured to gown by means of vertical tape strips, eye protection, full face shield and fit-tested N95/FFP2 or three respirators. Since all clothing worn should be waterproof and easy to decontaminate, operating room uniforms to be worn under PPE have been suggested. The use of disposable shoes covers is not recommended, due to the increased risk of contamination during the undressing phases [8].

One area of controversy relates to the use of powered air purifying respirators (PAPRs) instead of only N95/FFP masks for aerosol-generating procedures [6]. PAPRs are battery-powered blowers providing positive airflow through a filter, cartridge, or canister to a hood or face piece, selected on the basis of type and amount of airborne contaminant. 

PAPRs with high-efficiency particulate air (HEPA) or P100 filters have a greater level of respiratory protection than N95 masks. Additional advantages associated with the use of PAPRs include protection for the head and neck, guaranteed effectiveness even in the case of a beard, greater comfort in the case of prolonged use and less risk of displacement. Disadvantages are the greater difficulty of communication, the need for electricity (batteries) to guarantee the correct air flow in the hood and the need to decontaminate the reusable parts (batteries, helmet) [11,12]. Currently, WHO [6], ECDC [7] and ELSO [5] guidelines do not recommend the use of PAPR. Numerous experiences, however, report favorable results in their use, after proper training, in terms of safety and comfort for healthcare workers.

## 5. ECMO Treatment-Specific Features

ECMO is a highly skilled operation [2,3,4,5]. Considering the initial uncertainties about the 2019-nCov transmission, the invasiveness of the procedure, the possible long duration of exposure to patients and the possible recurrence of exposure by healthcare personnel specialized in ECMO positioning, we decided to provide healthcare workers in charge of ECMO missions with infection control precautions, consisting of a disposable waterproof suit, waterproof shoe covers and a hood.

Since the transmission mechanisms of SARS-CoV-2 infection are only partially known and given that healthcare workers wearing N95 masks were found to be infected after intervening with SARS patients [13], it was decided, in accordance with previous experiences [14], to implement the masks N95/FFP2-3 with PAPR Flyte (Stryker-Portage MI) with HEPA filters in all situations where high-risk procedures were required, such as connection to the ECMO circuit and transfer by ambulance. This is even in spite of the evidence from the literature suggesting that the use of PAPRs could be bulky and cumbersome, even impacting on the communication effectiveness [13,14]. However, this decision was made considering the high risk associated with the permanence of a high number of health-care workers in confined environments (such as the ambulance) for relatively long periods. In addition, the training phase, carried out wearing PPE and PAPR (see below), confirmed that neither manual nor communicative effectiveness was affected by the use of these devices.

## 6. Personal Protective Equipment Training

Personnel training is universally considered essential to ensure preparedness and success in dealing with a pandemic [15]. In our experience, after adequate PPE training, the use of all ECMO-related equipment had be practiced in simulation environment wearing PPE and PAPR. A multidisciplinary team was hence assembled to create a process with sterile attire and adapted from the WHO donning and doffing process [16]. The training procedure was limited to the donning and doffing phases, since no modification to the standard ECMO procedure was introduced. The procedures were simulated in a space dedicated to training, and therefore in the ward and in the ambulance, with the simulation of all personnel roles.

## 7. Our Center Donning and Doffing Procedures

**Figure 1:**
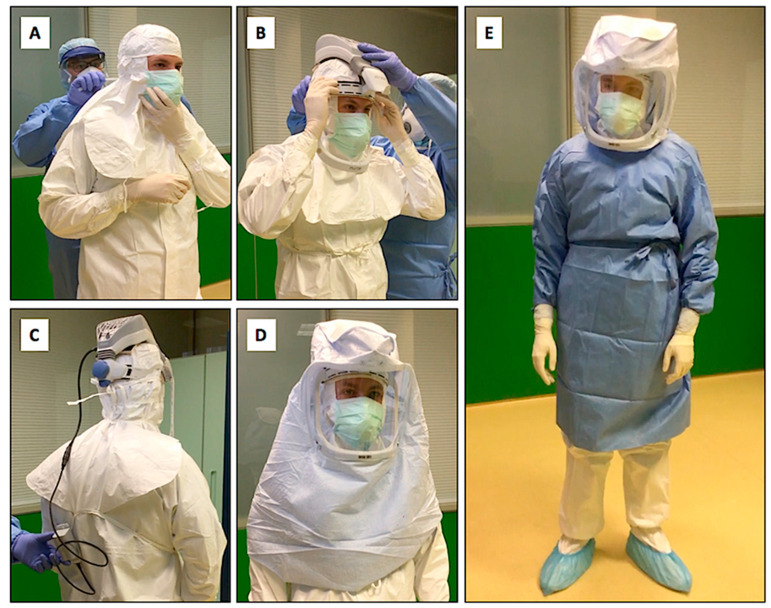
Enhanced droplet/airborne Personal Protective Equipment incorporating use of Powered Air Purifying Respirator (PAPR) for ECMO positioning. The healthcare staff wear impermeable disposable coveralls with impermeable overshoes and special waterproof hoods and surgical masks, which are worn over the headgear and FFP2-3 mask (**A**). The PAPR system is worn over the hood of the suit and PAPR blower unit, made by plastic helmet (**B**) with battery on belt (**C**) and covered by a hood with a filter material (**D**). Completed PPE includes a sterile gown and sterile gloves (**E**).

Since it was not possible to identify a real safe area for donning in most ICUs, it was prudently decided to carry out most of the PPE donning operations before leaving the ECMO center or outside the hospital in which the procedures for connecting to the ECMO were expected to take place, as illustrated in Table 1. Our enhanced droplet/airborne PPE procedure incorporates the use of PAPR with HEPA filters for ECMO positioning (Figure 1).

An additional intensivist was assigned the task of supervising each phase of the PPE management during the mission. Specifically, it was his responsibility: during the donning phase, to verify the complete adherence to the protocol; during the doffing phase, to check the integrity of the PPE removed. During the doffing phase, this support figure was also assigned the task of supporting healthcare workers in the removal of any contaminated PPE item (“dofficer” role), since the doffing procedure is particularly at risk of contamination for the healthcare worker. 

Our choice is supported by literature evidence. In fact, according to Verbeek et al., additional spoken instruction may lead to fewer errors in doffing (MD −0.9, 95% CI −1.4 to −0.4). Face-to-face instruction may reduce non-compliance with doffing guidance more (odds ratio 0.45, 95% CI 0.21−0.98) than providing folders or videos only [17], especially during the doffing procedure, that is particularly at risk of possible contamination for the healthcare professional, as emerged from the Ebola virus experience [18].

In cases where ECMO had to be positioned at another health Centre, the team should carry the PPE and PAPR resources with them to avoid the use of the not perfectly known equipment of another hospital. Personnel performing ECMO cannulation must wear sterile attire in addition to PPE, worn before starting the procedure in the patient′s room.

## 8. Preliminary Experience after Implementation of the Protocol

From 1 March to 31 December 2020, we used the procedure in 35 severe acute respiratory distress syndrome (ARDS) patients and one acute respiratory failure caused by neoplastic total tracheal obstruction, all suffering from pneumonia by 2019-nCoV, confirmed by the real-time polymerase-chain-reaction (RT-PCR) on at least one low respiratory tract specimen [19].

A protocol including the use of PAPRs, FFP 2-3 masks, protective suits, disposable sterile surgical gowns, and two pairs of sterile gloves (Figure 1) seems effective in protecting against the risk of contamination and allows full personnel ability of performing all ECMO-related procedures. All the involved staff (consisting of one anesthesiologist, cardiac surgeon, nurse and perfusionist) work in the patient room. Communication between the team members seems feasible with PAPRs. No major technical problems nor SARS-CoV-2 health care infection are reported in the involved staff.

Considering its feasibility, the approach proposed here has subsequently been extended to all procedures associated with a possible elevated risk of contamination when performed in COVID-19 patients.

## 9. Conclusions

Risks of contamination associated with the positioning and management of the ECMO in patients infected with 2019-nCov seem sustainable for the staff involved and safe for patients. 

Personnel training is essential and all ECMO-related equipment needs to be practiced in simulation environment wearing PPE and PAPRs.

## 10. Key Messages

- ECMO positioning deserves a specific effort in identifying appropriate PPE procedures being an invasive and long-lasting procedure, often repeated by experienced healthcare professionals
- A protocol including the use of powered air-purifying respirators, FFP 2-3 masks, protective suits, disposable sterile surgical gowns, and two pairs of sterile gloves is effective in protecting against the risk of contamination and allows full personnel ability of performing all ECMO-related procedures
- Personnel training is essential and all ECMO-related equipment need to be practiced in a simulation environment wearing personal protective equipment and powered air-purifying respirators.

## Figures and Tables

**Table 1:** Steps to put on/take off personal protective equipment (PPE) for ECMO procedures in patients with COVID-19.

Context	Put on	Context	Take off
Before leaving	- Satisfy primary needs - Remove jewelry/personal items, keep hair up and beard shaved - Perform hand hygiene - Put on the cap/head cover - Use the washable clogs with back strap - Put on the white impermeable overshoes - Place the white impermeable disposable coverall over the uniform (the second operator looks at the right closing of zip) - Put on the disposable overshoes ^a^	After ECMO connection—outside patient’s room	- Remove the external gloves - Remove the sterile gown - Perform hand hygiene by rubbing with antiseptic gel (on the internal gloves) - Remove the external disposable overshoes (with new non-sterile gloves or by the second operator) - Put on a new impermeable gown - Put on the second gloves - Check the Flyte battery ^c^ before leaving the ICU N.B. Final check of PPE by a second operator
At arrival (i.e., in the ambulance—out of the hospital)	N.B. Assistance of a second trained operator needed - Perform hand hygiene - Put on the FFP2/3 mask - Put on the white hood of the coverall ○ Fix the strips on the head ○ Fix the double strips of the mask and verify its adhesion to the face ○ Fix the inferior thorax strips anteriorly - Put on the sterile gloves ○ Fix the gloves on the sleeves of the coverall with tape strips	During transport	All the health-care team is wearing the full optional PAPR equipment. ^d^
Before entering patient’s room	- Adjust the helmet Flyte ^b^ to the head circumference - Put on the helmet Flyte ○ Check the size ○ Connect the battery ○ Verify functioning - Put on the helmet Flyte cover and be careful to fix it properly ○ Test the air flow - Put on the sterile gown - Put on the sterile gloves - N.B. Final check of PPE by a second operator	After arrival in in the ECMO-ICU	N.B. Assistance of a second operator, wearing proper PPE. Change gloves after each contaminated contact - Remove the external gown and gloves - Perform hand hygiene by rubbing with antiseptic gel (on the internal gloves) - While the first operator is sitting with his eyes closed, the second one, using movements for back to front and from top to bottom, unfastens and removes the Flyte helmet cover ○ Unfasten and disconnect the battery and place it in a designated container for its cleaning ○ Remove the Flyte helmet and place it in a designated container for its cleaning - Remove the white hood of the coverall - Remove the white coverall (the anterior zip should be opened by the second operator) and the internal gloves, simultaneously (because of the patch previously positioned) - Removal of the impermeable overshoes by the second operator (first operator sits on a plastic chair/support) - Remove the FFP2/3 mask and the cap/head cover - Remove the gloves and perform hand hygiene

^a^ The addition of the disposable overshoes is intended for mechanical protection only—i.e., to avoid traumatic damage of the impermeable overshoes. ^b^ We used the Flyte ^®^ Steri-Shield ^®^ personal protection system (Stryker). For more information: Available online: https://www.strykermeded.com/media/1082/flyte-sterishield-system-brochure.pdf (accessed on 30 April 2021). ^c^ For the management of the Flyte battery, follow manufacturer’s instructions. The battery replacement is performed by the second operator, wearing the proper PPE (impermeable gown—double gloves—goggles-face shield—head cover—FFP2-3 mask), opening the back of the first operator’s sterile gown and paying attention not to contaminate the battery. In the cleaning process, the battery and the Flyte helmet cannot be dipped into the water; pay attention to the electrical connections. ^d^ Support staff members (ambulance driver, stretcher bearers) wear coveralls, FFP2 masks, visors and gloves, upon arrival at target hospital until admission at our Hospital. List of abbreviations: PPE, personal protective equipment; ECMO, extracorporeal membrane oxygenation; COVID-19, coronavirus disease 2019; ICU, intensive care unit; FFP2/3, filtering face piece 2/3; PAPR, powered air-purifying respirator.

## Data Availability

Not applicable.
